# Endoscopic Topical Application (ETA) Therapy for Refractory Overactive Bladder: A First-in-Human Report

**DOI:** 10.7759/cureus.101143

**Published:** 2026-01-09

**Authors:** Takuya Sadahira, Masahiro Sugihara, Yosuke Mitsui, Toyohiko Watanabe, Motoo Araki, Masami Watanabe

**Affiliations:** 1 Department of Urology, Okayama University Graduate School of Medicine, Dentistry and Pharmaceutical Sciences, Okayama, JPN; 2 Center for Innovative Clinical Medicine, Okayama University Hospital, Okayama, JPN; 3 Department of Surgery, Nishi Fukuyama Hospital, Hiroshima, JPN; 4 Department of Urology, Okayama Urology Clinic, Okayama, JPN; 5 Department of Interdisciplinary Science and Engineering in Health Systems, Okayama University, Okayama, JPN

**Keywords:** bladder trigone, botulinum toxin, endoscopic topical application, new drug delivery systems, refractory overactive bladder

## Abstract

Refractory overactive bladder (OAB) remains a clinical challenge despite established therapies, such as anticholinergics, β3-agonists, and intradetrusor botulinum toxin (BTX). Emerging evidence suggests that sensory mechanisms within the bladder, including those involving the trigone where superficial afferent networks are present, may contribute to persistent urinary urgency and frequency in some patients. Although intradetrusor BTX injection is effective in selected patients, its impact on these superficial pathways may be limited because the injected drug predominantly distributes within the detrusor. Endoscopic topical application (ETA) therapy delivers BTX directly to the trigone under air cystoscopy, potentially providing targeted modulation of sensory hyperexcitability. We report a 72-year-old woman with long-standing refractory OAB who experienced only partial improvement with repeated intradetrusor BTX injections but achieved clinically meaningful symptom relief after ETA therapy. Nocturia, urgency, urgency urinary incontinence, and voided volume were improved, with no complications other than transient postoperative urethral pain. This case suggests that ETA therapy may represent a promising sensory-focused option for refractory OAB.

## Introduction

Overactive bladder (OAB) is defined by urinary urgency, typically accompanied by increased daytime frequency and nocturia, with or without urgency urinary incontinence, in the absence of infection or other identifiable pathology. Behavioral modifications and pharmacologic therapies, including anticholinergics and β3-adrenergic agonists, are commonly used in the initial management of OAB [1]. Patients who do not achieve adequate symptom control with these conservative and pharmacologic approaches are considered to have refractory OAB, for which intradetrusor botulinum toxin (BTX) injection is an established option [1]. Nonetheless, a significant proportion of patients continue to experience bothersome symptoms. Anatomical and neurophysiological studies suggest that sensory mechanisms, particularly those originating from the trigone where superficial afferent terminals are concentrated, may contribute to persistent urgency and frequency in a subset of patients [2-4]. This evidence also supports sensory-dominant mechanisms in a subset of refractory OAB patients, highlighting the trigone as a potential therapeutic target. Because intradetrusor BTX predominantly distributes within the detrusor muscle, its influence on these superficial sensory pathways may be limited. Based on this rationale, we developed an endoscopic topical application (ETA) therapy in which BTX is applied directly to the trigone under air cystoscopy, thereby maximizing exposure to superficial sensory structures. Here, we describe a patient with severe refractory OAB despite multiple prior treatments who experienced additional meaningful symptom improvement after ETA therapy, following partial response from multiple intradetrusor BTX injections.

## Case presentation

A 72-year-old woman with hypertension treated with antihypertensive medication presented with long-standing urgency urinary incontinence and severe urinary frequency. She had no allergies, surgical history, or family history of urological disease, and did not smoke or drink alcohol. At her initial presentation, she reported urinary urgency with nearly every void, urgency urinary incontinence requiring approximately 20 g/day of pad use, daytime frequency exceeding 15 voids per day, nocturia more than three times, and a mean voided volume of 120 mL (range: 80-160 mL). She was started on fesoterodine fumarate 4 mg but discontinued it after two weeks due to lower extremity edema. Mirabegron 50 mg was subsequently prescribed and initiated, then switched to vibegron 50 mg. Despite continued therapy, her symptoms persisted, with approximately 10 daytime voids, more than three nocturnal voids, urgency more than five times daily, urgency urinary incontinence more than five times daily, mean voided volumes of approximately 130 mL, and unchanged pad use.

She subsequently underwent her first intradetrusor BTX injection (100 U), which produced partial improvement: eight daytime voids, three nocturnal voids, increased mean voided volumes of 150 mL (range: 130-180 mL), reduced urgency episodes to three per day, and urgency urinary incontinence of one to three episodes daily, with intermittent pad use. Additional BTX injections (100 U) were administered on three subsequent occasions at approximately six-month intervals, resulting in stabilization of symptoms at eight daytime voids, two to three nocturnal voids, mean voided volumes of 165 mL (range: 110-200 mL), urgency occurring approximately three times daily, and one to two urgency urinary incontinence episodes daily. However, persistent nocturia, urgency, and incomplete control of incontinence remained problematic. After discussion of alternative treatment options as well as potential risks and benefits, the patient elected to undergo ETA therapy. ETA therapy was performed as an outpatient procedure in which BTX was applied securely to the mucosa around the bladder trigone using an endoscopy. This therapy aimed to relieve hypersensitivity of the bladder trigone area, which causes frequent urination and nocturia. 

**Figure 1:**
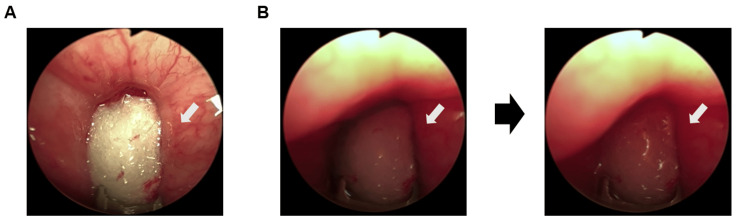
Endoscopic images during ETA therapy (A) Endoscopic view of the absorbent applicator pad on the midline trigone of the patient during the ETA therapy. The white arrows indicate bladder trigone. (B) Endoscopic view of the absorbent applicator pad with pooled BTX solution with indigo carmine around the pad is shown in the patient (left panel). After aspirating the pooled BTX, the applicator pad is almost free of the drug solution (right panel). The white arrows indicate indigo carmine. ETA: endoscopic topical application.

The instruments for the ETA therapy procedure consisted of an application catheter with a water-absorbent applicator pad bonded to its tip and a medical catheter introducer with a hemostasis valve. The application catheter worked as the injection tube with the applicator, and the catheter introducer worked as the aspiration tube. The principle of the drug irrigation and permeation system has been previously described [5]. The instruments are designed to deliver liquid drug endoscopically around the bladder trigone and neck and are placed in rigid cystoscopy before ETA therapy (Figure 1A). All components are commercially available, medical-grade devices, and drug delivery was standardized using a fixed volume and concentration of BTX with predefined injection followed by suction to ensure controlled dosing. Using a medical catheter tube, a water-absorbent pad, and medical adhesives, the application catheter was assembled under sterile conditions in the hospital. The applicator pad was bonded to the tip of the application catheter using adhesives. Given the patient's bladder trigone anatomy, the applicator pad was gently bent along its long axis to improve the fit of the trigone and neck during ETA procedures. The application catheter was inserted into the catheter introducer with a hemostasis valve, which had been prepared to an appropriate length to fit the cystoscopy, so that it could function as an aspiration tube to suck out excess drug solution from the applicator pad. Operators were experienced in cystoscopy and had received training on the system and the procedure before patient care.

Under local urethral anesthesia with 4% lidocaine, a rigid cystoscopy was introduced, and residual urine was drained. Air cystoscopic examination was performed by insufflating the bladder with approximately 100 mL of air to achieve adequate visualization while minimizing patient discomfort. After air cystoscopic examination, the applicator pad was placed over the midline trigone. The pad was then pushed down by the lever (Hebel) of the rigid cystoscopy and attached to the trigone and bladder neck. During treatment, BTX (100 U) diluted in 5 mL of solvent (saline: 4.5 mL; indigo carmine: 0.5 mL) was continuously applied to the mucosa of the bladder trigone and neck for 10 minutes using a 70-degree field cystoscopy with occasional air insufflation. BTX was injected into the applicator pad via the application catheter at 0.5 mL/min using a continuous drug injector. During the ETA procedure, excess liquid drug around the applicator pad was aspirated and removed through the catheter introducer (Figure 1B). The procedure was repeated on the target trigone, and subsequent applications using fresh BTX solution were performed to enhance drug permeation into the mucosa. The ETA procedure was initially directed to the midline trigone, then to the right and left ureteral orifices, to expand drug application around the trigone and neck and ensure coverage of the entire area. The procedure was well tolerated until completion of the ETA procedure, and the patient was discharged after confirmation of spontaneous voiding. Until the one-month follow-up, clinically meaningful improvement was observed: eight daytime voids, one to two nocturnal voids, urgency approximately once daily, reduced urgency urinary incontinence to one episode daily, and increased mean voided volumes of 200 mL (range: 150-270 mL). The only adverse event was transient urethral pain on postoperative day one, which resolved spontaneously. No urinary retention, urinary tract infection, or persistent hematuria occurred. The longitudinal clinical course and urinary symptom changes are summarized (Table 1).

**Table 1:** Clinical course and longitudinal urinary symptom outcomes Urinary symptoms are summarized over time across therapies showing partial stabilization with repeated intradetrusor BTX and additional improvement after ETA therapy. BTX: botulinum toxin, ETA: endoscopic topical application.

Time point	Intervention/status	Daytime frequency (voids/daytime)	Nocturia (voids/night)	Urgency (episodes/day)	Urgency urinary incontinence (episodes/day)	Pad use	Mean voided volume (mL)	Voided volume range (mL)	Notes/adverse events
Initial presentation (baseline)	Initial evaluation	>15	>3	>5	Nearly every void	20 g/day	120	80-160	Urgency with nearly every void
After anticholinergic therapy	Fesoterodine 4 mg (stopped after 2 weeks)								Discontinued due to lower extremity edema
On β3-agonist therapy	Mirabegron 50 mg → vibegron 50 mg (continued)	10	>3	>5	>5	Unchanged	130		
After intradetrusor BTX #1	BTX 100 U (first injection)	8	3	3	1-3	Intermittent	150	130-180	Partial improvement
After repeated intradetrusor BTX	BTX 100 U ×3 additional injections (approximately 6-month intervals)	8	2-3	3	1-3	Intermittent	165	110-200	Symptoms stabilized but residual nocturia/urgency
After ETA therapy (one-month follow-up)	ETA therapy (BTX 100 U)	8	1–2	1	1	Intermittent	200	150-270	Transient urethral pain on POD1; no retention/UTI/hematuria

## Discussion

**Figure 2:**
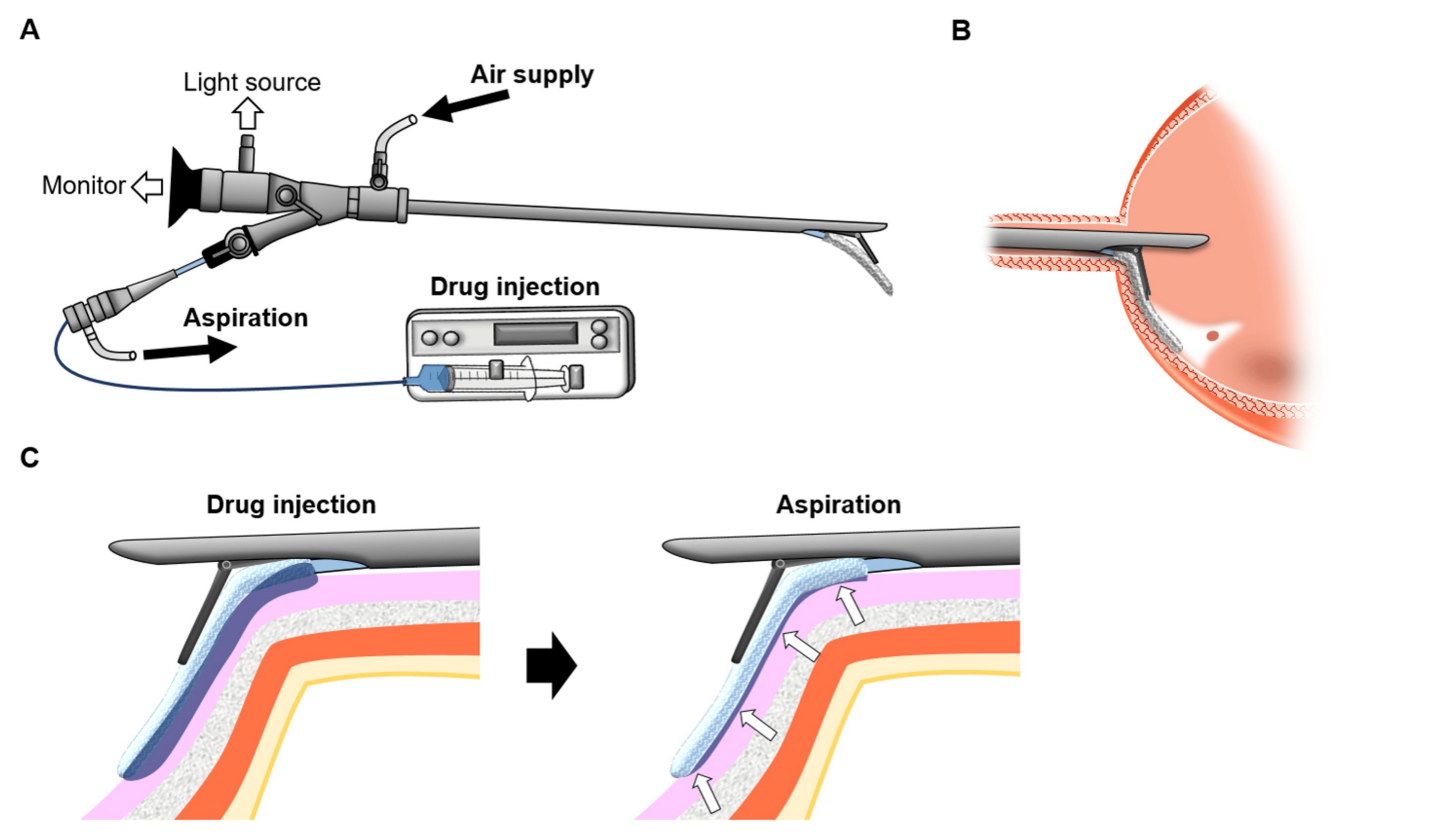
Schematic overview of the ETA therapy system and drug delivery mechanism (A) Schematic illustration of endoscopic topical application (ETA) therapy. ETA therapy under air cystoscopy is performed using rigid cystoscopy. The application catheter (diameter: 0.9 mm) is inserted into a catheter introducer (diameter: 1.5 mm) equipped with a hemostasis valve. A water-absorbent applicator pad (25 mm × 5 mm × 2 mm) is bonded to the tip of the application catheter. Botulinum toxin (BTX) solution is injected through the inner application catheter and absorbed by the applicator pad. The catheter introducer functions as an outer aspiration tube, enabling aspiration and removal of excess drug solution from around the applicator pad. (B) Schematic illustration of cystoscopic positioning of the absorbent applicator pad. During air cystoscopy, the applicator pad is pushed downward by the lever (Hebel) of the rigid cystoscope and applied directly to the trigone and bladder neck. (C) Schematic illustration of drug application using ETA therapy at the bladder trigone and neck. BTX solution containing indigo carmine is injected into the absorbent applicator pad via the application catheter. The administered solution is absorbed into the pad and transiently pools around it (left panel). After aspiration of excess drug solution through the catheter introducer, the pooled solution is removed (white arrows, right panel). These steps allow repeated delivery of fresh, undiluted BTX to the applicator pad and the attached target tissue. Image credits: Masami Watanabe and Takuya Sadahira.

This case illustrates the potential therapeutic value of ETA therapy in a patient with refractory OAB who had achieved only partial benefit from repeated intradetrusor BTX injection. Sensory-dominant OAB is believed to involve hyperexcitability of superficial afferent terminals located densely within the trigone and bladder neck region [3,4,6]. Intradetrusor BTX injections primarily affect deeper muscle layers and may have limited ability to modulate these superficial pathways. ETA therapy was designed to overcome this anatomical limitation by delivering BTX directly onto the trigonal mucosa. A schematic overview of the ETA therapy system and the proposed drug delivery mechanism is provided in Figure 2. Prior non-injection mucosal BTX strategies, such as intravesical liposomal BTX, demonstrated safety and short-term reductions in urgency and frequency, but may have limited ability to modulate deeper targets, potentially due to insufficient tissue penetration [7,8]. Effective mucosal BTX delivery appears to require prolonged, concentrated drug contact with the correct anatomical substrate.

**Figure 3:**
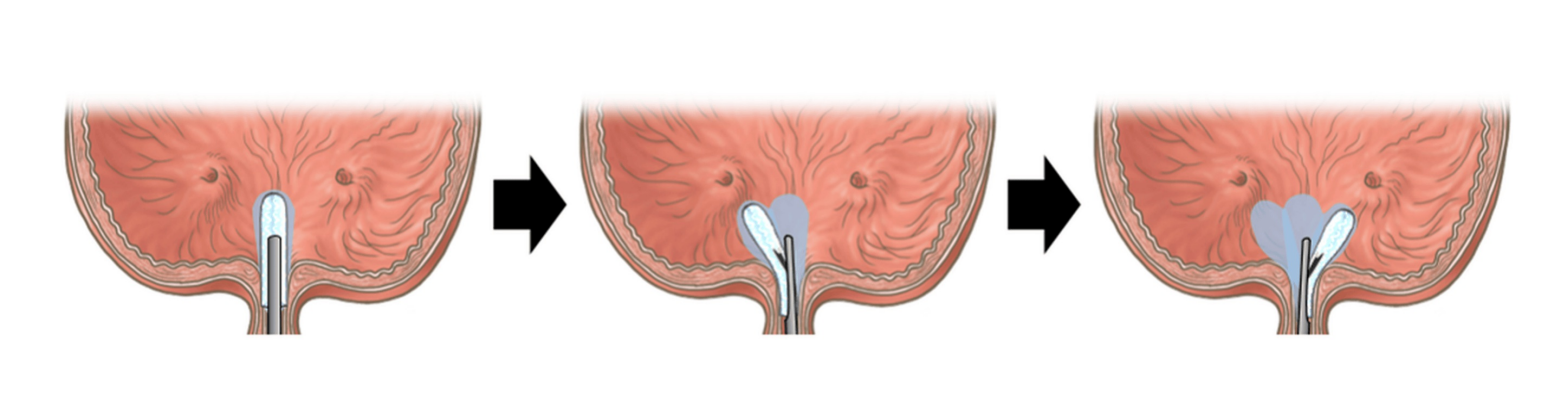
Conceptual diagram of trigonal BTX application during ETA therapy The ETA therapy is directed to the midline, then to the right and left ureteral orifices, to expand drug application around the trigone and bladder neck. The light blue markings indicate areas that have already been treated with the drug solution. ETA: endoscopic topical application, BTX: botulinum toxin. Image Credits: Masami Watanabe and Takuya Sadahira.

ETA therapy differs fundamentally from simple instillation. The aspiration procedure under air cystoscopy removes residual urine and mucus adherent to the mucosa, thereby preventing dilution and enabling sustained, undiluted contact between BTX and the trigonal epithelium. Although urine exposure could theoretically reduce the effective drug concentration through dilution and washout, particularly during the immediate post-administration period before sufficient mucosal uptake occurs, ETA therapy minimizes this effect by aspiration under air cystoscopy, allowing adequate contact time for uptake. The technique places BTX directly onto the trigone, where superficial afferent nerves are most densely distributed (Figure 3). It maintains contact with the mucosal surface long enough to promote penetration into epithelial sensory endings [2,3,9]. Importantly, this is achieved without needle penetration, avoiding trauma to the thin trigonal mucosa and submucosa. Collectively, ETA therapy may combine the safety advantages of non-invasive delivery with the anatomical specificity of injection-based treatments. The observed improvements in nocturia, urgency, and voided volume support the hypothesis that ETA therapy modulates aberrant afferent signaling and may overcome limitations of mucosal instillation methods. The absence of urinary retention suggests selective sensory modulation with preservation of detrusor contractility, an advantage particularly relevant for older adults or those at risk of impaired bladder emptying.

Limitations include the single-patient nature of this report and the short follow-up duration. Accordingly, factors such as urine exposure, effective contact time, and procedural optimization are addressed as important considerations for future investigation rather than determinants of efficacy in this feasibility-focused report. Optimal dosing, minimal effective dwell time, retreatment intervals, and the technically ideal body position and air bladder condition should be determined. Further prospective studies are needed to evaluate the long-term efficacy and safety of ETA therapy and to identify the ideal patient population. Recent mechanistic evidence supports the biological plausibility that BTX delivered via a mucosal route can penetrate the urothelium and modulate sensory pathways inadequately reached by intradetrusor injection [10]. BTX has been reported to suppress urothelial ATP release and increase nitric oxide production [2,11]. It has also been associated with downregulation of TRV1 and P2X3 receptors on suburothelial afferents [2,12]. This downregulation is predominantly observed within the urothelium and the superficial suburothelial layer [2,12]. These findings support the concept that BTX can diffuse into mucosal tissues and directly modulate abnormal sensory transduction. ETA therapy provides prolonged contact with a high concentration of BTX on the trigonal surface and may optimize mucosal uptake. In contrast, catheter-based instillation delivers BTX at a lower effective concentration, and urine dilution limits penetration [13]. These mucosal-level effects mechanistically account for the selective improvements in urgency and nocturia observed in this case, without causing detrusor underactivity.

## Conclusions

In this first-in-human feasibility-focused report, ETA therapy provided clinically meaningful symptom improvement for a patient with severe refractory OAB who had plateaued with conventional intradetrusor BTX injections. By specifically targeting the superficial sensory networks of the trigone, this needle-free approach modulates aberrant afferent signaling while preserving detrusor function. ETA therapy offers a promising sensory-focused and minimally invasive treatment option for refractory OAB, though larger clinical studies are essential to establish its long-term efficacy and safety.
